# Inflammatory Biomarkers in Febrile Seizure: A Comprehensive Bibliometric, Review and Visualization Analysis

**DOI:** 10.3390/brainsci11081077

**Published:** 2021-08-17

**Authors:** Ionela Maniu, Raluca Costea, George Maniu, Bogdan Mihai Neamtu

**Affiliations:** 1 Research Center in Informatics and Information Technology, Mathematics and Informatics Department, Faculty of Sciences, Lucian Blaga University, 550025 Sibiu, Romania; george.maniu@ulbsibiu.ro; 2 Research Compartment, Pediatric Clinical Hospital, 550166 Sibiu, Romania; ralucacosteadr@gmail.com; 3 Pediatric Neurology Department, Pediatric Clinical Hospital, 550166 Sibiu, Romania; 4 Medical Department, Faculty of Medicine, Lucian Blaga University, 550169 Sibiu, Romania; 5 Clinical Department, Faculty of Medicine, Lucian Blaga University, 550169 Sibiu, Romania; 6 Computer and Electrical Engineering Department, Faculty of Engineering, Lucian Blaga University, 550025 Sibiu, Romania

**Keywords:** febrile seizures, inflammatory biomarkers, literature mining, VOSviewer, clustering, visualization, bibliometric

## Abstract

Background: Inflammatory markers association with many diseases is the subject of many articles and reviews. This study presents a comprehensive bibliometric review and visualization analysis of inflammatory biomarkers (IB) in the context of febrile seizure (FS) patients. Methods: The study focused on IB in FS using (1) bibliometric analysis specific indicators and maps in order to analyze and present the network of authors, journals, universities, and countries, and (2) automated literature screening and unsupervised clustering approach for filtering and topic cluster identification. Results: We conducted a literature mining search on FS research, specifically IB in the context of FS, using the automated tools VOSviewer and Bibliometrix. Indices of the inflammatory response (in the context of febrile seizures) identified by the literature mining are (pro/anti-inflammatory) cytokines, such as interleukin IL-1β, IL-6, IL-8, I-10, IL-22, tumor necrosis factor (TNF-α), neutrophil-to-lymphocyte ratio (NLR), mean platelet volume (MPV), platelet count (PLT), and red blood cell distribution width (RDW). The resulted bibliometric maps and topic clusters offer a comprehensive overview, the status and leading trends on existing research of inflammatory biomarkers in FS. Conclusion: The analysis using bibliometrics and review with graphical presentations can be useful in investigating and (better) understanding the relationship between FS and IB.

## 1. Introduction

Febrile seizures triggered by infections can occur in children up to 5 years of age. Febrile seizures’ pathogenesis is based on cytokines release (pro- and anti-inflammatory) and genetic susceptibility to enhanced inflammation consisting of genetic variants of the pro-inflammatory and anti-inflammatory cytokines [1,2]. The link between inflammation and febrile seizures has been studied for more than 20 years [2]. The most relevant prospective case-control studies highlight the role of elevated levels of pro-inflammatory cytokines, triggering a rapidly rising fever, neuronal hyperexcitability and eventually the seizure event [2]. Among pro-inflammatory cytokines (released by activated microglia in the central nervous system or by monocytes, macrophages or T-lymphocytes in plasma) interleukin 1-beta (IL-1β), IL-6, and tumor necrosis factor (TNF)-α were found to be significantly elevated in children with febrile seizures [2]. The anti-inflammatory cytokines, such as IL-1 receptor antagonist (IL-1RA) and IL-10 along with anti-inflammatory cholinergic signals from the efferent vagus nerve, provide a negative feedback on the inflammation. Nevertheless, in susceptible patients, it seems that the negative feedback control is lost, and the elevated systemic levels of pro-inflammatory cytokine elicit the seizure event [1,2]. In clinical trials involving children with febrile seizures, it is expensive and not always available to validate specific patterns of the relevant pro-inflammatory cytokines. However, recent research reports highlight the importance of other inflammatory low-cost biomarkers, such as the neutrophil-to-lymphocyte ratio (NLR), platelet count (PLT) ratio, mean platelet volume (MPV) and platelet count, and red blood cell distribution width (RDW). It seems that NLR and MPV might synergistically determine the FS occurrence [2].

The cumulative number of research documents in many research fields, in general, and particularly in the febrile seizures (FS) field, is continuously increasing. Automated, interactive, flexible tools could be used by researchers to perform a systematic literature review and bibliometric analysis. Bibliometric analysis offers a quantitative and qualitative analysis of the analyzed publications. It is used in medical research but also in others research areas [3,4,5,6,7,8].

The current research aimed to perform a literature mining analysis on inflammatory biomarkers (IB) in the context of febrile seizure (FS). By analyzing the published documents and their citation and co-citation data, the current research themes presented in inflammatory biomarkers in FS research were identified. The specific objectives of this analysis were the following: (i) to assess the range of research topics; (ii) to identify which are the inflammatory biomarkers associated with febrile seizure discussed in the published literature; (iii) to identify who has driven this research; (iv) to assess what we can learn from this research; and (v) to guide investigators potential research directions and potential collaboration partners.

## 2. Materials and Methods

### 2.1. Search Methodology

**Figure 1:**
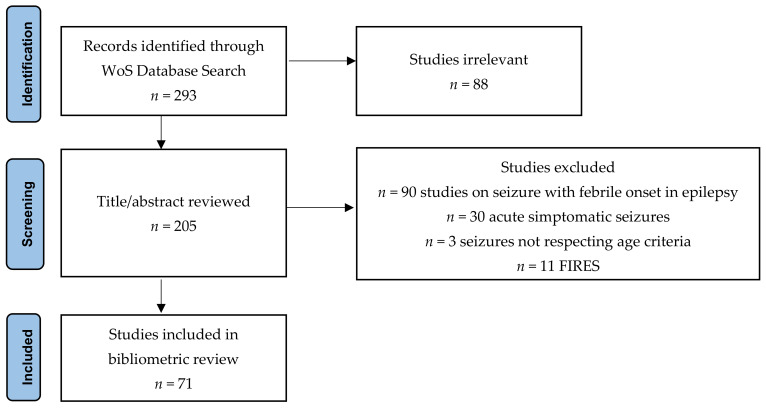
Flow diagram of document retrieval (according to the PRISMA statement).

In October 2020, we conducted a literature search on the Web of Science Core Collection (WoS) online databases to identify scientific contributions regarding febrile seizures and their association with inflammatory biomarkers. The search strategy included the terms “febrile seizures” alone and in combination with terms “marker” or “biomarker” and “inflamm*”. The asterisk was used to retrieve related/derivative words (inflammation, inflammatory, etc.). The search identified publications that contain the mentioned terms in their title or abstract or keywords. RC and BMN review the pool of documents selected by the automated review to reach a consensus on the inclusion of the topic-relevant ones. Discrepancies were sorted out with discussion. Papers that contained some of the search keywords but for which the major focus of the document was not related to inflammatory biomarkers in febrile seizure were excluded: studies on seizures with fever onset in epilepsy context; acute symptomatic seizures defined by (i) CNS (central nervous systems) infections (meningitis, encephalitis) and post infectious autoimmune encephalitis, (ii) dyselectrolytemia, (iii) TBI (traumatic brain injury), and (iv) others—sepsis, acute intoxications, seizures not respecting age criteria, FIRES (febrile infection-related epilepsy syndrome), irrelevant studies (psychiatric diseases, multiple sclerosis, stroke, TORCH infection, typhus, celiac disease, asthma, others) (Figure 1). No other restrictions/filters were considered for the research model (people/animal, article/review/letter/, English/French, etc.).

### 2.2. Data Extraction

Characteristics of each publication identified from the search include, besides the publication title, abstract and keyword, the following: authorship, document type, publication year, journal title, language, journal category, number of total citations. Data were extracted from WoS databases and exported as “tab-delimited text file”, “excel file”, “plain text” for additional processing.

The VOSviewer (visualizations of similarities, van Eck and Waltman, Leiden University, Leiden, Netherlands [9]) software was used to create network visualization maps (visualize and map bibliometric indicators). We analyzed the list of retrieved documents and bibliometric indicators for which ranking countries, institutions, journals, authors, keywords were presented [9,10,11,12,13,14]. We conducted term/items co-occurrence (maps) analysis based on the text data from the title and/or abstract and/or keyword of the publications, using the binary counting method (binary counting method vs. full counting method, the presence or absence of a term in a document matter vs. number of occurrences of a term in a document matter). In the term co-occurrence map, terms are represented by bubbles/circles, and some of them are labeled (to avoid overlapping). The number of publications in which the term was found is represented by the size of the circle. The relatedness of the terms is represented by the distance between the terms (the closer two terms are located to each other, the smaller the distance between the two terms; the larger the number of co-occurrences of the two terms—terms co-appear often, the stronger their relatedness) and curved lines between the terms (the larger the number of publications in which two terms were both found—co-occurrence, the stronger the relation/link between the terms and the thicker the line that links the two terms). Groups of terms which are strongly related to each other are represented using the same color (red, blue, green, etc.). When using the VOSviewer instrument, the following thresholds were set: a minimum of 5 occurrences for terms encountered in author keywords, all terms occurrences for terms encountered in the title. The historiographical citation network, co-citations network statistics, authors’ production over time, three-field plots were created using the Bibliometrix 3.1 package (Aria and Cuccurullo, University of Naples and University of Campania’s Luigi Vanvitelli, Italy, [15]) and RStudio 1.4.1717 environment (CRAN, https://cran.r-project.org/).

## 3. Results

The literature search using VOSViewer followed by document screening resulted in 71 publications indexed in WoS, the earliest published in 1996. Contributions in the field of febrile seizures and their association with inflammatory biomarkers came from 114 institutions located in 18 countries/regions. The most common type of retrieved documents were research articles (58; 82%) followed by reviews papers (8; 11%). The analysis of research areas of the retrieved documents showed that 53 articles were in neuroscience and neurology, 21 in pediatrics, 7 in immunology, 3 in pharmacology and pharmacy and in psychiatry, while 2 were in areas such as science and technology, internal medicine, allergy and others.

**Figure 2:**
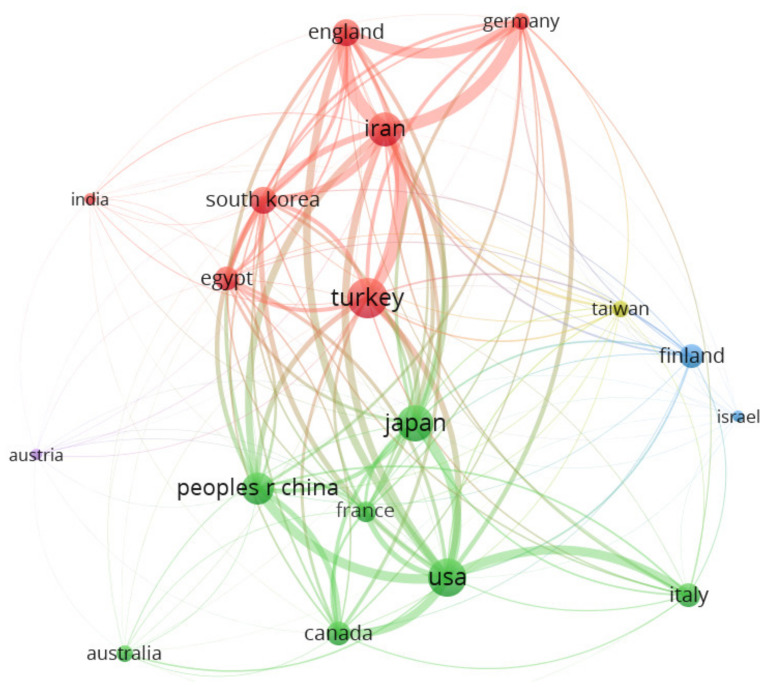
VOSviewer network visualization map (type of analysis: bibliographic coupling, weights—documents) of countries/regions involved in inflammatory markers in febrile seizures research. Of the 18 countries/regions, 7 had at least 5 publications. There were 5 clusters of countries/regions: 1—7 items, cluster 2—7 items, 3—2 items, 4—1 item, 5—1 item.

International collaboration in the context of the analyzed studies was highlighted by constructing visualization maps of countries and institutions involved in inflammatory markers in febrile seizures research studies. The collaboration analysis of countries/regions is presented in Figure 2, wider lines indicating stronger collaboration. The five most productive countries in terms of produced documents are Turkey (11), U.S.A. (10), Japan (9), Iran (8), and China (7). The country/region co-authorship network highlights the research activities: (i) U.S.A. had collaborations with Italy (3), Japan (1), Canada (1), China (1), Germany (1), and England (1); (ii) Iran had collaborations with Germany (1), and England (1); (iii) Germany had collaborations with U.S.A. (1), England (1), and Iran (1); and (iv) Canada had collaborations with U.S.A. (1), and China (1). Studies from Japan, Italy, Finland, Israel appeared earlier (2006–2008), while studies from Iran, South Korea, and China appeared later (2016). The country with the highest total link strength (indicating that it participated in the most collaborations with other countries worldwide) was Iran (1771) followed by U.S.A. (1644), England (1143), Turkey (1106), and China (1038). The highest number of citations belonged to U.S.A. (789), followed by Italy (532), Japan (278), Finland (220), and Turkey (165). The statistics and ranks of the countries are presented in Table 1.

**Figure 3:**
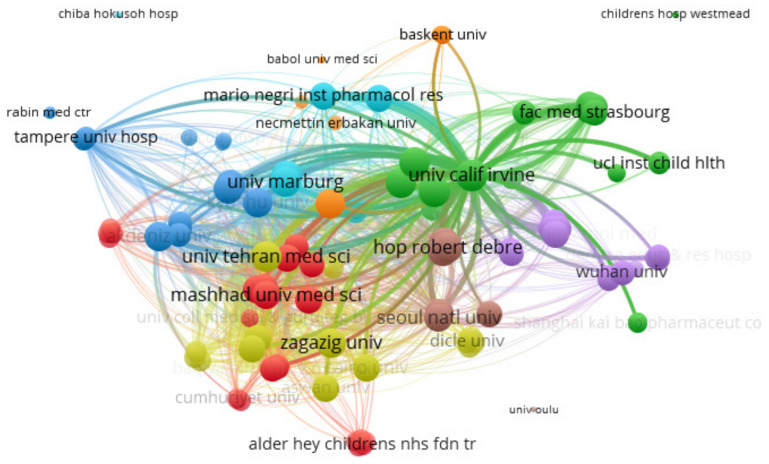
VOSviewer network visualization map (type of analysis: bibliographic coupling, weights—links) of institutions involved in inflammatory markers in febrile seizures research. Of the 114 organizations, 8 had at least 3 publications. There were 9 clusters of institutions: 1—21 items, 2—18 items, 3—16 items, 4—14 items, 5—13 items, 6—10 items, 7—8 items, 8—7 items, 9—3 items.

The collaboration analysis of institutions is presented in Figure 3. The top five organizations by produced documents are Tehran Univ. of Medical Sciences (5 publications), Mario Negri Institute for Pharmacological Research (4 publications), University of California, Irvine (UCI) (4 publications), Ehime Univ. (4 publications), and University of Social Welfare and Rehabilitation Sciences (4 publications); the top five by total link strength are Tehran Univ. of Medical Sciences (3142), University of Social Welfare and Rehabilitation Sciences (1804), Universal Scientific Education and Research Network (USERN) (1480), Marburg Univ. (1428), and Sheffield Univ. (1428); the top five by total citations are Mario Negri Institute for Pharmacological Research (532), Scripps Research Institute (500), University of California, Irvine (UCI) (413), Tampere Univ. (220), and Yamaguchi Univ. (112).

**Figure 4:**
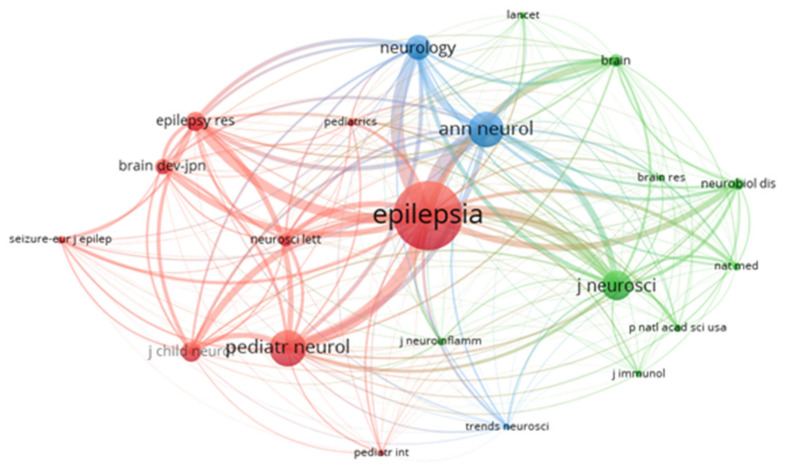
VOSviewer network visualization map (type of analysis: co-citation, weights-citation) of journals. Larger bubbles indicate higher co-citations.

The most productive journals (Table 2) were the following: *Epilepsia* (IF2019 = 6.04, Q1) with 8 publications in the field, followed by *Pediatric Neurology* (IF2019 = 2.89, Q1), *Seizure-European Journal of Epilepsy* (IF2019 = 2.52, Q3), *Brain and Development* (IF2019 = 1.50, Q4), and *Journal of Child Neurology* (IF2019 = 1.71, Q3). There were 579 co-cited journals (two journals are co-cited if there is a third journal that cites both journals [16]) and 21 had at least 20 citations (Figure 4). The most co-cited journals (Table 1) were the following: *Epilepsia* with 213 citations followed by *Pediatric Neurology*, *Annals of Neurology* (IF2019 = 9.03, Q1), *Journal of Neuroscience* (IF2019 = 5.67, Q1), and *Neurology* (IF2019 = 8.77, Q1). *Epilepsia* had an active co-citation relationship with *Annals of Neurology* (663 citations), *Neurology* (541 citations), *Pediatric Neurology* (513 citations), *Journal of Neuroscience* (457 citations), and *Epilepsy Research* (423 citations).

**Figure 5:**
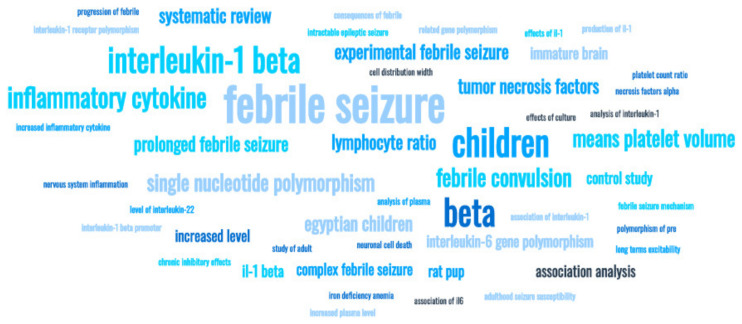
Inflammatory markers in the context of febrile seizure research (visualization using Word Cloud generator from Monkey Learn).

**Figure 6:**
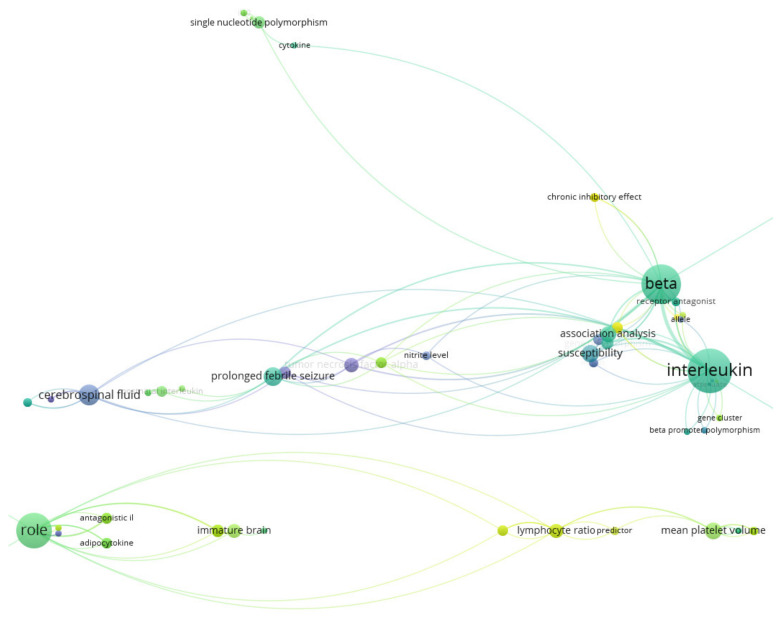
Inflammatory markers in the context of febrile seizure research—visualization using VOSviewer -network visualization map (type of analysis: co-occurrence, weights—links) of terms from the title of the document corpus. Co-occurrence and linkages among the terms according to their time of appearance are presented (blue color—early appearance, yellow color—later appearance).

The most frequently encountered terms in author keywords of the retrieved documents were the following: febrile seizure (s), cytokine (s)—mentioned in more than 20 cases, interleukin IL-1 beta, [2,17,18,19,20,21,22,23,24], (gene) polymorphism [1,25,26,27,28,29,30,31,32,33], inflammation/inflammatory—mentioned in more than 10 cases (each), interleukin-6 (7 cases [21,26,29,30,34]) and others. Indices of the inflammatory response (in the context of febrile seizures) identified by the literature mining are the following: (pro/anti-inflammatory) cytokines, such as interleukin (IL)-1β, IL-6, IL-8, IL10, IL-22, tumor necrosis factor (TNF)-α), neutrophil-to-lymphocyte ratio (NLR), mean platelet volume (MPV), platelet count (PLT), and red blood cell distribution width (RDW). Their visual representation is depicted in Figure 5 and Figure 6. The term map visualization from Figure 5 was created by analyzing (using natural language processing techniques) the term from the titles of the retrieved documents. Of the 119 words (all words from the titles) we selected, for the visualization map, the one related to inflammatory biomarkers. In the upper part of Figure 6, we observed terms dealing with inflammatory biomarkers, such as cytokines/interleukins (expensive, not always available biomarkers), while on the other side are biomarkers from blood: NLR, MPV, PLT, RDW (low-cost, available biomarkers).

**Figure 7:**
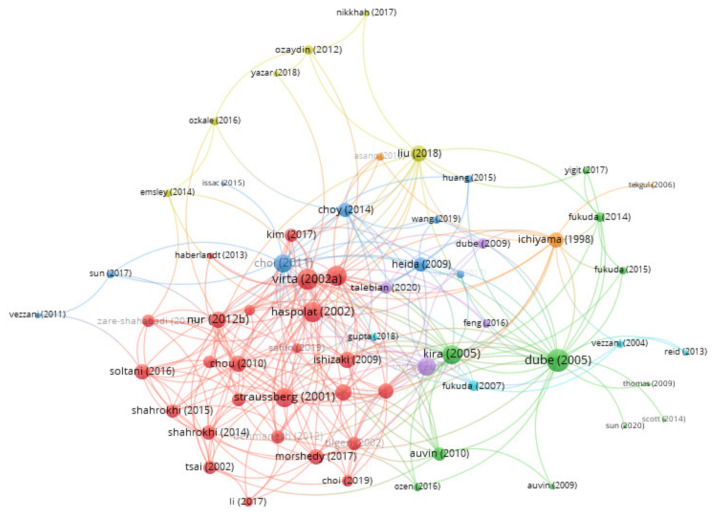
VOSviewer network visualization map (type of analysis: citation, weights—links, normalization method (fractionalization)) of publications. Of the 71 publications, 62 had at least 1 citation. A citation link is a link/connection/relation between two items (one item cites the other) [12].

The citation relationship between the publications is presented in Figure 7. Papers with a higher number of citations are considered (i) a reflection of the popularity and (ii) influencers of the research in the field/scientific community [35]. Among the most cited publication in the researched field are those referring to cytokines. One publication is the article “Interleukin-1 beta contributes to the generation of experimental febrile seizures”, written by Dube, C, Vezzani, A, Behrens, M, Bartfai, T, Baram, TZ, which was published in Annals of Neurology in 2005 and has 297 citations in WoS [36]. Another publication is the article “Increased plasma levels of pro- and anti-inflammatory cytokines in patients with febrile seizures”, written by Virta, M, Hurme, M, Helminen, M, which was published in *EPILEPSIA* in 2002 and has 119 citations in WoS [37]. In addition, the last review (with meta-analysis) refers to cytokines: “Cytokine levels in febrile seizure patients: A systematic review and meta-analysis”, written by Kwon, A.; Kwak, B.O.; Kim, K.; Ha, J.; Kim, S.J.; Bae, S.H.; Son, J.S.; Kim, S. N; Lee, R. and published in *Seizure-European Journal of Epilepsy* in 2018 [2]. Only a small number of publications have attempted to address the relationship between inflammatory biomarkers from blood (NLR, MPV, PLT, RDW) and febrile seizures [38,39,40,41,42,43]. Most of these publications are part of the green-yellow cluster (the north-west region on the network visualization map of publications, Figure 6). This cluster (with nine items) is composed mostly of authors of Turkish and Iranian origin and presents a visible differentiation from the rest of the (clusters) publications. Top twenty most cited publications (listed in Figure 6 and Figure 7) are presented in more detail in Table 3.

**Figure 8:**
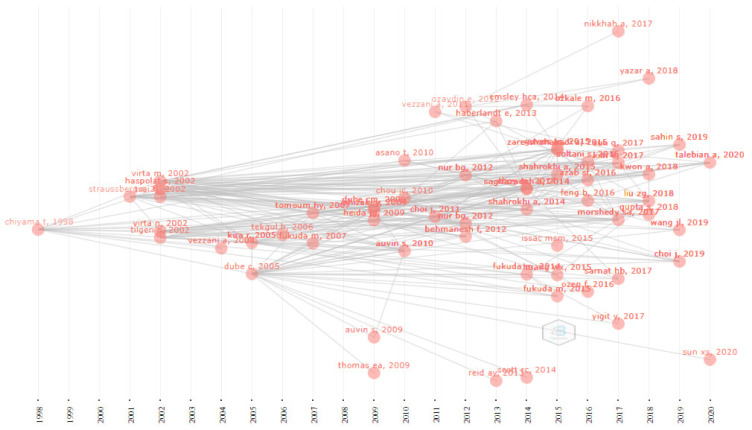
Bibliometrix historiographic citation network.

Using Bibliometrix, a historiograph of publications is displayed in Figure 8, and network statistics (in terms of centrality, cohesion, impact) are presented in Table 4. The centrality measure—betweenness centrality (based on leading eigenvalues clustering method)—highlighted as main “influencers” (have high betweenness centrality) the documents written by Dube [36], Virta [37,44], and Haspolat [24]. These publications are core/pivotal nodes that make connections to other publications within the network. The historiograph from Figure 8 provides an overview of the trends and evolution of the field by the flow of links between the cited publications (from the left, the older one) and the citing publications (from the right, the recent one).

**Table 3:** Top 20 most cited documents.

Node (Author) Year (Ref.)	Paper Title	Journal	Citations	Links
Dube et al., 2005 [36]	Interleukin-1 beta contributes to the generation of experimental febrile seizures	Annals of Neurology	309	29
Vezzani et al., 2011 [22]	IL-1 receptor/Toll-like receptor signaling in infection, inflammation, stress and neurodegeneration couples hyperexcitability and seizures	Brain behavior and immunity	203	2
Virta et al., 2002 [37]	Increased plasma levels of pro- and anti-inflammatory cytokines in patients with febrile seizures	Epilepsia	125	25
Ichiyama et al., 1998 [45]	Tumor necrosis factor-alpha, interleukin-1 beta, and interleukin-6 in cerebrospinal fluid from children with prolonged febrile seizures—Comparison with acute encephalitis/encephalopathy	Neurology	112	14
Dube et al., 2009 [46]	Febrile seizures: Mechanisms and relationship to epilepsy	Brain and Development	93	7
Virta et al., 2002 [44]	Increased frequency of interleukin-1 beta (-511) allele 2 in febrile seizures	Pediatric Neurology	90	18
Choi et al., 2011 [21]	Increased levels of HMGB1 and pro-inflammatory cytokines in children with febrile seizures	Journal of neuroinflammation	76	19
Heida et al., 2009 [47]	The role of interleukin-1 beta in febrile seizures	Brain and Development	72	12
Haspolat et al., 2002 [24]	Interleukin-1 beta, tumor necrosis factor-alpha, and nitrite levels in febrile seizures	Journal of child Neurology	71	22
Straussberg et al., 2001 [48]	Pro- and anti-inflammatory cytokines in children with febrile convulsions	Pediatric Neurology	50	21
Kira et al., 2005 [33]	Genetic susceptibility to simple febrile seizures: Interleukin-1 beta promoter polymorphisms are associated with sporadic cases	Neuroscience Letters	49	20
Dale et al., 2009 [49]	Cerebrospinal fluid neopterin in pediatric neurology: a marker of active central nervous system inflammation	Developmental medicine and child neurology	48	0
Chou et al., 2010 [31]	Interleukin (IL)-1 beta, IL-1 Receptor Antagonist, IL-6, IL-8, IL-10, and Tumor Necrosis Factor alpha Gene Polymorphisms in Patients with Febrile Seizures	Journal of clinical laboratory analysis	38	15
Tilgen et al., 2002 [50]	Association analysis between the human interleukin 1 beta (-511) gene polymorphism and susceptibility to febrile convulsions	Neuroscience letters	38	11
Ishizaki et al., 2009 [32]	Interleukin-10 is associated with resistance to febrile seizures: Genetic association and experimental animal studies	Epilepsia	36	15
Vezzani et al., 2004 [51]	Functional role of proinflammatory and anti-inflammatory cytokines in seizures	Recent advances in epilepsy research	32	4
Saghazadeh et al., 2014 [23]	Proinflammatory and anti-inflammatory cytokines in febrile seizures and epilepsy: systematic review and meta-analysis	Reviews in the neurosciences	31	24
Tomoum et al., 2007 [52]	Plasma interleukin-1 beta levels in children with febrile seizures	Journal of child neurology	31	15
Fukuda et al., 2007 [20]	Interleukin-6 attenuates hyperthermia-induced seizures in developing rats	Brain and Development	31	7
Auvin et al., 2009 [53]	Inflammation in rat pups subjected to short hyperthermic seizures enhances brain long-term excitability	Epilepsy Research	31	2

**Figure 9:**
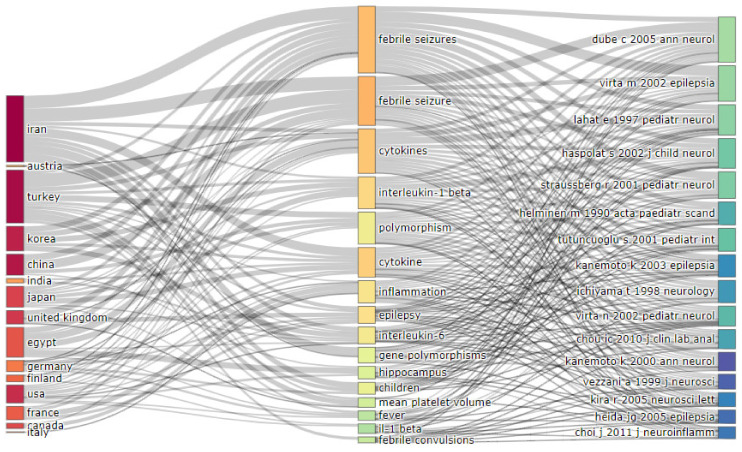
Bibliometrix three-fields plot presenting the relationship between meta-data such as countries, keywords, references.

When analyzing the relationship between three publications meta-data—countries/institutions, keywords, references (Figure 9)—it is possible to observe that researchers from particular countries/institutions used/analyzed particular keywords/biomarkers and considered particular reference sources (citations traceability/patterns in relation with terms/biomarkers).

## 4. Discussion and Conclusions

Although pathogenic mechanisms of febrile seizures remain unclear, experimental studies demonstrate that inflammation and inflammatory mediators are the main causes and propagators of febrile seizures [36]. New trends are targeting cytokines as more sensitive, yet more expensive biomarkers, in exploring febrile seizures, as evidenced by our bibliometric tool search.

In this study, we used the bibliometric methodology to perform a literature-driven analysis in order to identify research directions and trends in our study context.

U.S.A. appeared in the top positions in citations, bibliographic coupling and co-citation analysis, indicating the impact and high-ranking level of this country. The country’s bibliographic coupling analysis, reflecting how they are connected in terms of the bibliographic common literature, indicate two clusters: the first cluster includes seven countries (colored with green): U.S.A., Japan, China, Italy, Canada, France and Australia. The second cluster includes also seven countries (colored with red): Turkey, Iran, South Korea, England, Egypt, Germany and India. *Epilepsia*, the most productive and co-cited journal, was the influential journal in the field. Besides this journal, *Pediatric Neurology* was also among the top productive and co-cited journals.

Pro-inflammatory cytokines involved in febrile seizures’ pathogenesis frequently reported in the research papers were (i) IL-1β, IL-6, IL-8, IL-12, IL-22, (TNF)-α, (ii) IFN-γ(important activator of macrophages involved in autoimmune disorders), (iii) transforming growth factor beta (TGF-β) suspected to trigger astrocytes’ activity leading to EEG modified patterns toward seizures [2,17,54,55], and (iv) high mobility group box 1 protein (HGMB1) secreted by activated macrophages and monocytes [21]. However, the key interleukin involved in febrile seizures pathogenesis was IL-1β. HMGB1, IL-6, TNF-α positively correlated with elevated levels with IL-1 β [2,21]. We highlight the fact that the network of terms from Figure 6, clusters generated by the software, presents the IL-1 β node and links it both with the gene polymorphism node, and with other cytokines nodes along with single nucleotide polymorphisms-SNPs node. Genetic variants are highly referred in these research papers. We found gene polymorphism for IL-1β [1,19,25,32,33], HMGB1, IL-6 [26,29,30], TNF-α [27] and TGF-β [28].

The most frequently encountered anti-inflammatory cytokines in the studied papers were IL-1RA, IL-10 and IFN-β. IL-1RA [18] counteracts IL-1 β, IL-10 inhibits TNF-α and IFN-γ, while IFN-β is a marker of Toll-like receptor-3 activation, suggesting a host response to viruses. It modulates the expression of both pro-and anti-inflammatory agents in the brain, reducing the blood–brain barrier permeability to inflammatory cells. It is acknowledged in the current literature that therapy with IFN-β alleviates neuroinflammation. In febrile seizures however, it did not show a statistical significance compared to the control group, according to the reports of Sahin S. et al. [54]. The genetic variants for anti-inflammatory cytokines mentioned in the literature are mainly related to IL-1RA [31] and IL-10 [32].

The bibliometric analysis provided little evidence of studies related to common inflammatory biomarkers sensitivity and specificity in febrile seizures (e.g., total leukocytes count, neutrophils, lymphocytes and monocytes count). However, inflammatory indices, such as NLR, PLT, the NLT/PLT ratio, MPV, and RDW, seem to be useful biomarkers in this respect. These indices are easier and less expensive to evaluate in clinical practice than cytokines [38,39,40]. Recent research papers explore this possibility. It seems that NLR and MPV might synergistically determine the FS occurrence [2]. Moreover, NLR and MPV might differentiate simple versus complex febrile seizures [41,42].

Considering the network visualization of terms and network visualization of publications, we can highlight the fact that, in relation to inflammatory biomarkers in febrile seizures, there are two clusters of publications: the first one includes publications that address the relationship between cytokines/interleukins (expensive, not always available biomarkers) and febrile seizure, while the second one includes publications which address the relationship between biomarkers from blood (NLR, MPV, PLT, RDW—low-cost, available biomarkers) and febrile seizures. Among the most cited publications in the researched field are those referring to cytokines [31,32,54], and only a small number of publications have attempted to address the relationship between the aforementioned indices from blood and febrile seizures [41,42]. Moreover, there are studies in the literature that show IL’s action on the liver, thus leading to increased C-reactive protein [56]. Due to this fact, the testing of this anti-inflammatory (available) marker could be useful in febrile seizures. In addition, the synthesis of the abovementioned interleukin is triggered by certain biological stimuli, represented by the endotoxins of Gram-negative bacteria, often involved in urinary tract infections [57].

The analysis of publications, keywords, and references offers an overview of the trends and popular topics on the state of febrile seizure and inflammatory biomarkers. Using bibliometric analysis could guide researchers toward journals and authors associated with the field of their research interest. Furthermore, it could provide clues and facilitate links to other research centers with the same field of interest, as we identified five main institutions (Tehran Univ. of Medical Sciences, Mario Negri Institute for Pharmacological Research, University of California, Irvine (UCI), Ehime Univ., and University of Social Welfare and Rehabilitation Sciences) with activity related to inflammation and febrile seizures. Among the limitations of the current research, we mention the fact that we analyzed a sample of related publications (not claiming to cover all related literature), only from the WoS database. The results are based on a keyword (from title, abstract, keyword plus) analysis, and not on the publications full-text analysis. Researchers should also consider combining different bibliometric, review and meta-analysis tools [58,59,60,61,62,63,64,65] for their literature mining research. Going deeply with bibliometric analysis, considering network and cluster metrics, multivariate methods, comparisons between networks generated using different keywords, developing content analysis, meta-analysis, etc., could offer better insights in understanding the research topics.

## Figures and Tables

**Table 1:** Statistics by countries.

Rank	Documents	Citations	Total Link Strength		
			Co-Citations	Citations	Bibliographic Coupling
1	Turkey (11)	U.S.A. (789)	U.S.A. (8)	U.S.A. (92)	Iran (1771)
2	U.S.A. (10)	Italy (532)	England (3) Germany (3) Italy (3)	Turkey (87)	U.S.A. (1644)
3	Japan (9)	Japan (278)	Canada (2) Iran (2) China (2)	Iran (82)	England (1143)
4	Iran (8)	Finland (220)	Japan (1)	Japan (69)	Turkey (1106)
5	China (7)	Turkey (165)		Finland (53)	China (1038)
6	South Korea (5) England (5)	England (128)		South Korea (51)	Japan (998)
7	Finland (4) Egypt (4) Italy (4) Canada (4)	Canada (126)		China (46) Egypt (46)	Germany (978)
8	France (3)	South Korea (94)		Italy (38)	South Korea (821)
9	Germany (2) Taiwan (2) Australia (2)	Australia (73)		England (33)	Canada (718)
10	India (1) Austria (1) Israel (1)	Germany (69)		Taiwan (30)	Egypt (629)

**Table 2:** Top productive and co-cited journals.

Rank	Productive Journals	Count/FPY	IF2019/JCR	IB	Rank	Co-Cited Journals	Citations/TLS	Cluster/Items	IF2019/JCR
1	Epilepsia	8 1998	6.04 Q1	cytokines IL 10	1	Epilepsia	213 4961	1 9	6.04 Q1
2	Pediatric Neurology	5 1996	2.89 Q1/Q2	IL 6, IL 4, IL 1 beta, cytokines	2	Pediatric Neurology	113 2390	1 9	2.89 Q1/Q2
2	Seizure-European Journal of Epilepsy	5 2012	2.52 Q3	cytokines, IL 10, mean platelet volume	3	Annals of Neurology	109 2777	3 3	9.03 Q1
2	Brain and Development	5 2006	1.50 Q4	IL 1, IL 6	4	Journal of Neuroscience	92 2111	2 9	5.67 Q1
3	Journal of Child Neurology	4 2002	1.71 Q3/Q4	IL 1, TNF	5	Neurology	79 2363	3 3	8.77 Q1

FPY—first publication year, IF—impact factor, Q—quartile in category, JCR—journal citations reports, IB—inflammatory biomarker, TLS—total link strength.

**Table 4:** Bibliometrix co-citation network statistics.

Node	Cluster	Betweenness	Closeness	Page Rank	LCS	GCS	LCS/GCS ratio	NLCS	NGCS
Dube 2005	1	190.112	0.013	0.024	28	309	9.06	2.05	2.58
Virta 2002	1	151.126	0.013	0.024	24	125	19.2	1.54	1.78
Haspolat 2002	2	108.1	0.013	0.022	19	71	26.76	1.22	1.01
Virta 2002	2	93.012	0.012	0.021	16	90	17.78	1.03	1.28

LCS—local citations, impact of a document in the analyzed corpus of documents, GCS—global citations, impact of a document in the whole bibliographic database (in our case WoS), NLCS—Normalized Local Citation Score, calculated by dividing the actual count of local citing items by the expected citation rate for documents with the same year of publication, NGCS—Normalized Global Citation Score [9].

## Data Availability

Not applicable.
